# Banti's Syndrome in an Adult Male: A Case Report

**DOI:** 10.7759/cureus.25521

**Published:** 2022-05-31

**Authors:** Ahmad R. Khan, Muhammad Hayyan Wazir, Salma Waqar, Rizwan Ullah, Aiysha Gul

**Affiliations:** 1 Internal Medicine, Hayatabad Medical Complex Peshawar, Peshawar, PAK; 2 Obstetrics and Gynaecology, Mardan Medical Complex, Mardan, PAK

**Keywords:** ascites, pancytopenia, idiopathic portal hypertension, massive splenomegaly, non cirrhotic portal hypertension, banti syndrome

## Abstract

Banti’s syndrome is a chronic congestive enlargement of the spleen leading to the destruction of blood cells resulting in pancytopenia. It is also associated with cirrhosis and ascites along with symptoms of pancytopenia such as infection, bruising, weakness, and fatigue. Multiple factors such as hepatitis B infection, coagulation abnormalities and exposure to arsenic, etc. may also cause Banti’s syndrome. Clinical evaluation with blood profile along with use of imaging studies such as MRI and splenic venography is utilized for the determination of Banti’s syndrome. In this report, we present a 29-year-old diabetic male who presented with abdominal distention, right leg cellulitis, fever, and a past history of hematemesis and melena. On examination, distended abdomen showed marked splenomegaly with ascites (positive shifting dullness and fluid thrill). Also, the left leg was warm, swollen, and tender to the touch. Complete blood count showed decreased WBC, RBC, Hb, with peripheral smear negative for malarial parasites. Ultrasound scan of abdomen and pelvis was done illustrating massive splenomegaly with pelvic dilation and ascites.

## Introduction

Banti’s syndrome is characterized by massive splenomegaly, pancytopenia due to hypersplenism, and portal hypertension without any liver pathology. It is a diagnosis of exclusion when all causes of portal hypertension, splenomegaly, and pancytopenia are excluded. This syndrome was first described by Guido Banti, an Italian professor, in the late 19 century. It is known by different terminologies worldwide. It is called non-cirrhotic portal fibrosis in India [1], idiopathic portal hypertension (IPH) in Japan, hepatoportal sclerosis in the United States of America [2], and Banti’s syndrome in Europe. Although this syndrome rarely occurs globally, it is more common in developing countries like India and Pakistan and affects middle-aged individuals more frequently. Compared to Japan, in India, males are more commonly affected than females [3].

In this syndrome, the spleen is primarily affected. Other features include: varices seen on endoscopy, cytopenia of one or more cell lines, elevated portal hypertension with prominent portosystemic shunts, absence of liver cirrhosis, patent hepatic veins, and normal liver function tests. In the late stages, this syndrome is complicated by rupture of varices and life-threatening hemorrhage [4]. In Pakistan, a case series was conducted on 37 patients with portal hypertension; 18 were found to have IPH [5]. Another case was reported at the Agha Khan Hospital, Karachi, Pakistan [6]. Here, we report a case of a 29-year-old male who presented with a history of fever and left foot cellulitis for the past three days, abdominal pain and distension, and a history of melena and hematemesis. 

## Case presentation

A 29-year-old male, a known type 2 diabetic, having a history of one episode of hematemesis and melena 15 years ago, presented to us with abdominal distention, right leg cellulitis, and fever. There was no history of jaundice, diarrhea, easy bruising, cough, altered sensorium, chest pain, palpitations, breathlessness, orthopnea, or paroxysmal nocturnal dyspnea (PND). 

On general physical examination, the patient was of thin physique, pale-looking, lying on the bed in a supine position, and well oriented in time, place, and person. He was febrile (100 F). His pulse was 94/ min, regular, with normal volume and character. His blood pressure was 120/70 mmHg. There was no icterus, clubbing, cyanosis, or lymphadenopathy. Jugular venous pressure (JVP) was normal. On abdominal examination, massive splenomegaly was present, palpable edge beyond umbilicus (grade 4). The liver was palpable. The abdomen was distended with positive fluid shift, and the skin over the abdomen was thin and shiny with no dilated veins, scars, and no visible pulsations. Other systems' review was normal. Liver function tests and renal function tests were within the normal range. Prothrombin time, activated partial thromboplastin time, and international normalized ratio (INR) were normal. Urine routine examination was normal. Inflammatory markers were slightly deranged. HBsAg, anti-HIV, and anti-HCV were negative.

**Figure 1:**
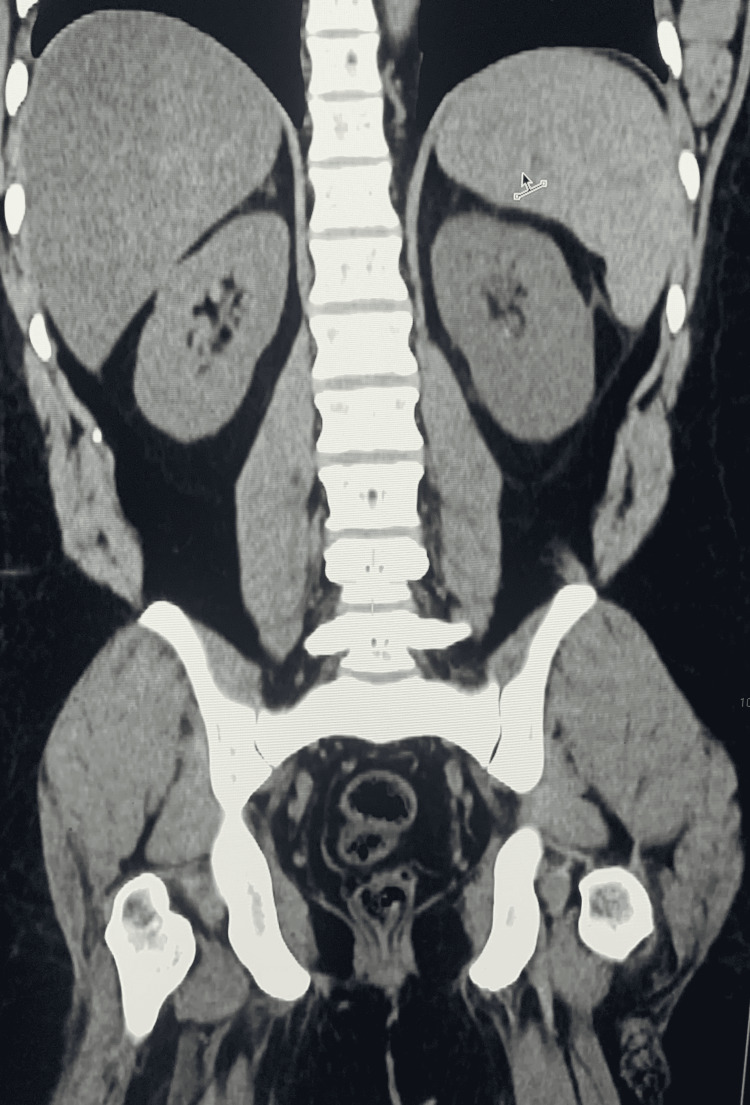
CT scan of abdomen (coronal view without contrast) shows: liver is enlarged with diffuse fatty infiltration; gallbladder is normal without any intraluminal calculus; adrenals and pancreas are unremarkable; spleen is enlarged and measures 26 cm with homogenous texture.

**Figure 2:**
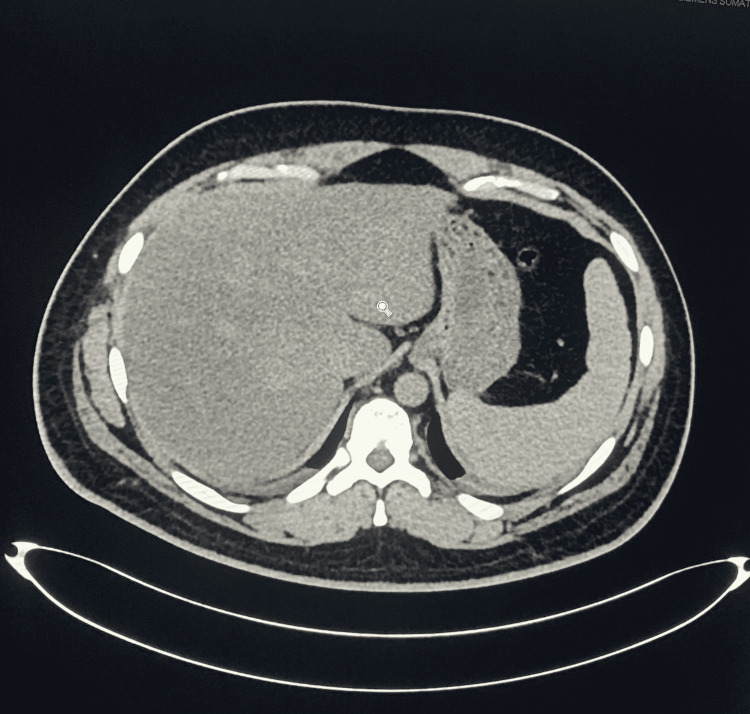
CT scan of abdomen (axial view without contrast).

Complete blood count showed a WBC count of 3700/mm^3^, RBC count of 2.72 million/mm^3^, hemoglobin 7.6 gm%, mean corpuscular volume 81.3, platelets 25,000. In differential leukocyte count (DLC), lymphocytes were 4.5%, neutrophils were 90%, eosinophils were 0.5%, monocytes were 5%, and basophils were 0 %. Peripheral smear showed no malarial parasites. Ultrasonography and CT scan of the abdomen and pelvis (Figures 1, 2) showed massive splenomegaly measuring 26 cm with homogenous texture. The portal vein was dilated 16mm, with a Vmax of 15 cm/sec. Gross abdominopelvic ascites were present.

## Discussion

Banti’s syndrome (also known as Banti’s disease, hypersplenism, idiopathic congestive splenomegaly, or IPH) is a chronic congestive enlargement of the spleen, resulting in blood cell destruction. Symptoms of Banti's syndrome include weakness, fatigue, anemia, and splenomegaly. 

With time, hematemesis and melena, associated with the condition, can make the anemia more profound. Although cirrhosis of the liver can eventually develop, splenomegaly is the primary symptom and ascites can develop. Coronavirus disease 2019 (COVID-19) has also been reported to present as Banti’s Syndrome [7]. Patients with Banti's syndrome often have symptoms related to pancytopenia, including the risk of severe infections, bruising, and fatigue. The etiology of Banti’s syndrome is multifactorial including the role of infectious agents (hepatitis B virus), coagulation abnormalities, and toxic metal exposure, including arsenic [6,8].

There is some evidence to support immunologic abnormalities in this syndrome [9]. Nayyar et al. found that total T cells and cytotoxic T cells were diminished. Since tumor necrosis factor (TNF) is involved in fibrosis, there is evidence that increased soluble TNF receptors I and II, which are found in this syndrome [10], explain the fibrosis around the portal vein. Furthermore, since TNF causes upregulation of vascular cell adhesion molecule-1 (VCAM-1), increased VCAM-1 is also observed in such patients [11,12].

The major symptoms in patients with Banti's syndrome are GI hemorrhage and splenomegaly, which were found in our patient. endoscopic sclerotherapy and endoscopic variceal band ligation can be performed in the emergency setting. Beta-blockers for primary prophylaxis should be given for portal hypertension even in the absence of cirrhosis. Splenectomy may be indicated for recurrent bleeding and severe anemia requiring multiple blood transfusions [13]. With successful treatment of variceal hemorrhage, patients with Banti’s syndrome can have an excellent prognosis, with five-year survival up to 100%. 

## Conclusions

Banti's syndrome is a chronic congestive enlargement of the spleen leading to the destruction of blood cells resulting in pancytopenia. Knowing about its rare incidence, one shall be considerate about the approach in diagnosing this disease among other differential diagnoses and proper management necessary to intervene illness.
